# Sodium Oxalate-Induced Acute Kidney Injury Associated With Glomerular and Tubulointerstitial Damage in Rats

**DOI:** 10.3389/fphys.2020.01076

**Published:** 2020-08-25

**Authors:** Larissa de Araújo, Juliana Martins Costa-Pessoa, Mariana Charleaux de Ponte, Maria Oliveira-Souza

**Affiliations:** Laboratory of Renal Physiology, Department of Physiology and Biophysics, Institute of Biomedical Sciences, University of São Paulo, São Paulo, Brazil

**Keywords:** sodium oxalate, crystalline nephropathy, acute kidney injury, glomerular and tubulointerstitial injury, albuminuria, inflammation

## Abstract

Acute crystalline nephropathy is closely related to tubulointerstitial injury, but few studies have investigated glomerular changes in this condition. Thus, in the current study, we investigated the factors involved in glomerular and tubulointerstitial injury in an experimental model of crystalline-induced acute kidney injury (AKI). We treated male Wistar rats with a single injection of sodium oxalate (NaOx, 7 mg⋅100 g^–1^⋅day^–1^, resuspended in 0.9% NaCl solution, i.p.) or vehicle (control). After 24 h of treatment, food and water intake, urine output, body weight gain, and renal function were evaluated. Renal tissue was used for the morphological studies, quantitative PCR and protein expression studies. Our results revealed that NaOx treatment did not change metabolic or electrolyte and water intake parameters or urine output. However, the treated group exhibited tubular calcium oxalate (CaOx) crystals excretion, followed by a decline in kidney function demonstrated along with glomerular injury, which was confirmed by increased plasma creatinine and urea concentrations, increased glomerular desmin immunostaining, nephrin mRNA expression and decreased WT1 immunofluorescence. Furthermore, NaOx treatment resulted in tubulointerstitial injury, which was confirmed by tubular dilation, albuminuria, increased Kim-1 and Ki67 mRNA expression, decreased megalin and Tamm–Horsfall protein (THP) expression. Finally, the treatment induced increases in CD68 protein staining, MCP-1, IL-1β, NFkappaB, and α-SMA mRNA expression, which are consistent with proinflammatory and profibrotic signaling, respectively. In conclusion, our findings provide relevant information regarding crystalline-induced AKI, showing strong tubulointerstitial and glomerular injury with a possible loss of podocyte viability.

## Introduction

Oxalate is an end product of hepatic metabolism of glyoxylate, amino acids and carbohydrates (Gambardella and Richardson, 1977; Poore et al., 1997; Knight et al., 2011). In addition, exogenous oxalate is supplied by a diet composed especially of green leafy vegetables, seeds and roots (Noonan and Savage, 1999). Intestinal oxalate absorption is predominantly passive and paracellular. In addition, transcellular transport of oxalate is mediated by SLC26 anion exchangers expressed on both apical and basolateral membranes of intestinal epithelial cells (Efe et al., 2019; Knauf et al., 2019). In healthy humans, the plasma oxalate levels are fairly low (1–6 μmol/L) (Kasidas and Rose, 1986; Hoppe et al., 2009). Oxalate is primarily excreted by the kidneys via glomerular filtration and tubular secretion, the last one being mediated by the SLC26A anion exchanger expressed in the basolateral membrane (Robijn et al., 2011) and the Cl^–^/oxalate exchanger SLC26A6 expressed mainly in the brush-border membrane of proximal tubule cells (Bergsland et al., 2011; Knauf et al., 2019) and in the apical membrane of distal nephron cells (Kujala et al., 2005).

Studies have demonstrated that an internal oxalate imbalance results in hyperoxaluria (Pfau and Knauf, 2016), which predisposes to the formation of renal stone. In fact, it is a common and multifactorial disease, occurring in 8% of the population and considered related to environmental factors and diseases such as metabolic syndrome, diabetes mellitus, obesity and kidney disease progression (Meydan et al., 2003; Carbone et al., 2018; Waikar et al., 2019). Hyperoxaluria leads to urinary calcium oxalate (CaOx) supersaturation, resulting in the formation of crystals in the kidney parenchyma and tubules, consistent with nephrolithiasis or nephrocalcinosis (Sayer et al., 2004).

During the acute supersaturation phase, CaOx complexes are the most common type of kidney stone, which was initially referred to as type 2 crystalline nephropathy (Mulay et al., 2018). Under this condition, tubular injury is associated with crystals and/or Tamm–Horsfall protein (THP) complexes, apoptotic and inflammatory responses consistent with acute kidney injury (AKI), in addition to the risk for recurrence and/or chronic kidney disease (CKD) progression (Lorenz et al., 2014; Pfau and Knauf, 2016; Fox et al., 2018; Mulay et al., 2018; Efe et al., 2019). However, clinical evidence and experimental models of ischemia-reperfusion have revealed that frequently, the recovery of renal function after AKI is incomplete and accompanied by proteinuria, tubular injury and glomerular filtration rate (GFR) decline, leading to end-stage renal disease (ESRD) (Hingorani et al., 2009; Basile et al., 2012). Furthermore, other studies using animal models of ischemia-reperfusion and glomerulosclerosis have identified podocyte injury as an etiological factor of progressive proteinuria and kidney function decline (Hara et al., 2015; Chen et al., 2019).

In the clinical context of nephrolithiasis, the investigation of factors involved with crystalline-induced AKI is essential to understand the relationship between AKI events and CKD progression with or without recurrence. Compared to humans, rodent present a special resistance to crystal retention (Khan and Glenton, 2010). However, the animal models can provide a deeper insight into the molecular mechanisms involved in the renal injury. In view of these findings, in the current study, we used a crystalline-related AKI model induced by a single injection of sodium oxalate solution (NaOx). We sought to explore the factors involved in glomerular and tubulointerstitial injury associated with the intrarenal CaOx crystal formation in this rodent model.

## Materials and Methods

### Animal Study Design

Male Wistar rats aged 60 days (*n* = 15, weighing 150–250 g) were obtained from the animal care facility of the Department of Physiology and Biophysics, Institute of Biomedical Sciences, the University of São Paulo (São Paulo, Brazil). The experimental protocols were conducted in accordance with the ethical standards approved by the Institutional Animal Care and Use Committee of the University of São Paulo (Protocol no. 9276140518). All animals were housed at the department facility under standard conditions (constant temperature of 22°C, 12:12-h light-dark cycle, 60% relative humidity, fed standard rat chow and water *ad libitum*). The animals were randomly allocated into the following two groups: (1) control rats (*n* = 7), which received a single vehicle injection of 237 μL/100g of 0.9% NaCl solution (i.p.); (2) sodium oxalate-treated rats (*n* = 8), which received a single injection of NaOx (7 mg⋅100 g^–1^⋅day^–1^) (Khan et al., 1992; Khan and Glenton, 2010), resuspended in 237 μL/100g of 0.9% NaCl solution (i.p.) (Synth, Diadema, SP, Brazil). Just after the injection, the animals were placed individually in metabolic cages (Techniplast, Milan, Italy) for 24 h. The food (g/day) and water (mL/day) intake as well as urine output (μL/min) were analyzed. At the end of 24 h of treatment, the animals were anesthetized with ketamine (75 mg/kg i.p.) and xylazine (4 mg/kg i.p., Virbac, Jurubatuba, São Paulo, Brazil), placed on a warm table to maintain body temperature and prepared surgically for cannulation of the distal aorta using a PE-50 catheter (Clay Adams Company, Inc., Parsippany, NJ, United States) for blood sample collection followed by kidney perfusion with PBS (0.15 M NaCl containing 10 mM sodium phosphate buffer, pH 7.4) at 20 mL/min using a peristaltic perfusion pump, BP600 (Milan Scientific Equipment, Colombo, PR, Brazil) as previously described (Casare et al., 2016; de Ponte et al., 2017). Euthanasia was performed by exsanguination. One kidney was isolated, removed, weighed and snap frozen for further quantitative PCR and protein expression studies. The remaining kidney was fixed in 4% paraformaldehyde solution, dehydrated and embedded in paraffin for morphological studies.

### Plasma and Urine Analysis

Urine sediment (30 μL) was examined under a light microscope (Eclipse 80i, Nikon, Tokyo, Japan) to confirm the occurrence of crystals. Plasma osmolality was measured using an osmometer (Precision Systems, Natick, MA, United States) and sodium concentration was determined by flame photometry (9180 Electrolyte Analyzer; Roche, Basel, Switzerland) as previously described (Casare et al., 2016; de Ponte et al., 2017). Plasma urea and creatinine as well as urine creatinine levels were evaluated using colorimetric tests (Labtest, Lagoa Santa, MG Brazil). The creatinine clearance was calculated using the following formula: [*C* = (*Urine_*Cr*_⋅V*)*/Plasma_*Cr*_*], where C is clearance, Cr is creatinine, and V represents urinary flow. The urinary albumin excretion was determined using a SilverQuest Silver Staining Kit (Thermo Fisher Scientific, Waltham, MA, United States) by the modified Oakley method (Oakley et al., 1980). Briefly, urine samples (volume corresponding to 5 μg creatinine) from the metabolic cages were separated by SDS polyacrylamide gel electrophoresis (10%). Next, silver staining was performed on the gel, and albumin bands were identified using a molecular weight marker (bovine serum albumin – BSA, 66 kDa). The bands were analyzed by optical densitometry using ImageJ software [National Institutes of Health (NIH), Bethesda, MD, United States].

### Total Renal Tissue mRNA Expression Studies

The isolated kidney was cut into two sections, which were quickly frozen and pulverized in liquid nitrogen. As previously described (de Ponte et al., 2017) and summarized here, frozen kidney sections were exposed to TRIzol LS Reagent (Life Technologies, Carlsbad, CA, United States) for RNA isolation. Then, 2 μg of total RNA was used to obtain cDNA (High-Capacity cDNA Reverse Transcription Kit; Life Technologies) and real-time PCR was performed using a StepOnePlus (Life Technologies) machine and TaqMan assay system (Life Technologies). The following TaqMan probes were used: nephrin (*NPHS1*), Rn00674268_m1; kidney injury molecule-1 (Kim-1) (*Havcr1*), Rn00597703_m1; Ki67 (*Mki67*), Rn01451446_m1; monocyte chemoattractant protein-1 (MCP-1) (*Ccl2*), Rn00580555_m1; interleukin 1 beta (*Il1b*), Rn00580432_m1; nuclear factor kappa B 1 (*Nfkb1*), Rn01399572_m1; α-SMA (*Acta2*), Rn01759928_g1; and glyceraldehyde-3-phosphate dehydrogenase (*Gapdh*), Rn01775763_g1 (reference gene). All qPCRs were performed using 20 ng cDNA and all samples were assayed in duplicate. The comparative cycle threshold (2^Δ^ ^Δ^ ^*Ct*^) method was used for data analysis. The data were normalized to *Gapdh* expression and are shown as the fold change relative to the control group.

### Immunoblotting Studies

Total protein was extracted from the remaining kidney section using ice-cold PBS with protease inhibitors (Roche Brazil, São Paulo, Brazil) and centrifugation (3,000 × *g* for 10 min at 4°C). As previously described (Costa-Pessoa et al., 2014; Gonçalves et al., 2018) and summarized here, immunoblot analysis was performed on 50-μg protein aliquots resolved by 4 or 10% SDS-PAGE. Then, the protein samples were transferred to a polyvinylidene fluoride (PVDF) membrane. The blots were blocked and incubated with the following primary antibodies: mouse anti-THP (1:1000), goat anti-megalin (1:1000) (Santa Cruz Biotechnology, SC, CA, United States) and mouse anti-β-actin (1:5000, Abcam, Cambridge, United Kingdom). A horseradish peroxidase-conjugated secondary antibody (Jackson ImmunoResearch Laboratories, Baltimore, MD, United States) was used and the blots were treated with enhanced chemiluminescence (ECL) reagent (GE HealthCare, Aurora, OH, United States). The bands were quantified by optical densitometry using ImageJ software (National Institutes of Health) and protein expression was analyzed relative to the endogenous control β-actin. The values are presented as protein expression relative to the control group.

### Morphological Studies and Immunostaining

As previously described (Thieme and Oliveira-Souza, 2015) and summarized here, fixed 4-μm kidney sections were deparaffinized for histological studies. Next, the histological sections were stained with hematoxylin and eosin (HE) and examined under a light microscope (Eclipse 80i, Nikon) to evaluate tubular morphology, interstitial conditions and tubular crystal formation. Additionally, deparaffinized 4-μm kidney sections were subjected to immunohistochemical staining with rabbit anti-desmin (1:200, Abcam), mouse anti-CD68 (ED-1) (1:50, AbD Serotec, Oxford, United Kingdom) and mouse anti-THP (1:400, Santa Cruz Biotechnology) primary antibodies. Non-specific protein binding was first blocked by incubation with 10% goat serum in TBS + BSA 1% for 60 min and then the sections were incubated with primary antibodies overnight at 4°C. The reaction products were detected using avidin-biotin-peroxidase complex (Vector Labs, Burlingame, CA, United States) and the sections were counterstained with methyl green (Amresco, Ohio, United States), dehydrated and mounted with Permount (Fisher Scientific, Fair Lawn, NJ, United States). The immunostained proteins were analyzed using a computerized morphometry program (NIS-Elements, Nikon) with 20 and 40× objectives. THP staining was qualitatively analyzed using a light microscope (Eclipse 80i, Nikon). The number of CD68-positive infiltrating cells was counted in 30 fields (107172, 99 μm^2^) and the mean of the control and treated groups were compared. Glomerular desmin staining was quantified using Aperio ImageScope software version 12.3.2 (Leica Biosystems, Buffalo Grove, IL, United States) and protein staining is expressed as the total intensity of the positive signal. Fluorescence measurements were performed as previously reported with minor modifications (Cardoso et al., 2018). Four-micrometer deparaffinized kidney sections were blocked with 3% BSA in PBS for 1 h at room temperature before being incubated overnight at 4°C with rabbit anti-WT1 (1:200) and goat anti-megalin (1:200) primary antibodies (Thermo Fisher Scientific). Next, the kidney sections were washed three times with PBS, incubated with Alexa Fluor 594-conjugated *F*(ab′) goat anti-rabbit (1:200) and Alexa Fluor 488-conjugated *F*(ab′) rabbit anti-goat (1:200) secondary antibodies (Thermo Fisher Scientific) for 1 h at room temperature in the dark, and mounted with Fluoroshield (Sigma Aldrich). Megalin staining was analyzed using a Zeiss LSM 510 confocal microscope equipped with a 63× objective and laser excitation at 488 nm. Tubular megalin fluorescence signal was quantified using the ImageJ (National Institute of Mental Health, Bethesda, MD, United States) and the equation: *Corrected total cells fluorescence* = *integrated density −* (*area of selected cells × means fluorescence background*). The numbers of positive tubules were counted in three areas per animal and the means of the control and treated groups were compared. For WT1, kidney sections were analyzed using a fluorescence microscope (Eclipse 80i, Nikon, Tokyo, Japan) equipped with a 40× objective and laser excitation at 546 nm for Alexa Fluor 594 and 405 nm for DAPI. The number of WT1-positive cells and DAPI-positive cells were counted in 10 glomeruli per animal. Then, the WT1/DAPI ratio was calculated and the mean of the control and treated groups were compared. All morphological analyses were performed blindly by one independent investigator.

### Statistical Analysis

Comparisons between two groups were performed using a non-parametric Mann–Whitney test using GraphPad Prism Software (GraphPad Software, Inc., San Diego, CA, United States). The results are expressed as the mean ± standard error of the mean (SE) and *p* < 0.05 was considered significant.

## Results

### Metabolic Parameters

As shown in Table 1, NaOx treatment did not change the food and water intake, urinary flow, kidney weight, plasma sodium concentration or osmolality in comparison to the control group.

**TABLE 1:** Metabolic parameters.

Parameters	CTL (*n* = 6–7)	NaOx (*n* = 7–8)	*p-*value
Food intake (g/day)	47.004.51	48.171.26	0.8765
Water intake (mL/day)	15.003.10	15.141.26	0.6435
Urinary flow (μL/min)	16.511.89	13.611.39	0.2810
Kidney weight/body weight (mg/g)	3.840.12	4.160.16	0.1520
Plasma sodium (mEq/L)	135.801.86	133.301.40	0.2646
Plasma osmolality (mOsmol/kg H_2_O)	308.805.44	317.704.57	0.1352

*Values are mean ± SE, number of animals (n) per group is indicated in parenthesis*.

### Kidney Function

**FIGURE 1:**
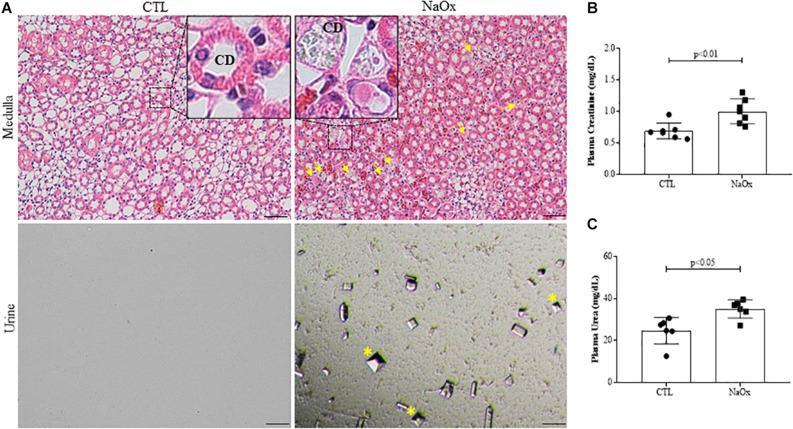
Representative photomicrographs of renal tissue illustrating the medullar area and urinary sediment in control and treated rats. Kidney sections (4-μm thick) were stained with hematoxylin and eosin (HE). **(A)** Standardized images were captured using a morphometric program (NIS-Elements, Nikon) with a 20× objective, and magnified images captured with a 40× objective are shown at the top. The arrows indicate crystal formations and protein casts in collecting ducts (CD), and the asterisks indicate urinary CaOx crystals. The effects of NaOx treatment on plasma creatinine **(B)** and urea **(C)** levels. The values are the means ± SEs (*n* = 6–8). Bar = 100 μm. CTL, control; NaOx, sodium oxalate.

To confirm the establishment of the experimental model of NaOx-induced acute crystalline nephropathy, hematoxylin and eosin staining was performed and the staining revealed medullary casts and CaOx crystals, which were also confirmed by urinary sediment analysis (Figure 1A). Plasma levels of creatinine and urea were significantly increased after acute NaOx treatment (Figures 1B,C and Table 2). In addition, urinary creatinine concentration and creatinine clearance were decreased in comparison to those in the control group (Table 2), although urinary flow remained unchanged as showed in Table 1.

**TABLE 2:** Differences among experimental group.

Parameters	CTL (*n* = 6–7)	NaOx (*n* = 6–8)	*p-*value
Plasma creatinine (mg/dL)	0.690.05	1.000.07	0.0041**
Urinary creatinine (mg/dL)	42.372.01	31.561.43	0.0159*
Creatinine clearance (mL/min)	1.080.06	0.560.05	0.0007***
Plasma urea (mg/dL)	24.712.61	35.031.77	0.0152*

**Parameters**	**CTL (*n* = 5–6)**	**NaOx (*n* = 5–6)**	***p*-value**

Glomerular desmin expression (intensity signal)	279264673	775666440	0.0079**
WT-1/DAPI (ratio)	0.490.01	0.420.02	0.0143**
Nephrin (*Nphs1*) mRNA expression *(Fold Change)*	1.000.05	1.270.06	0.0152*

**Parameters**	**CTL (*n* = 6–7)**	**NaOx (*n* = 6–8)**	***p*-value**

Alb/Cr ratio (AU)	48.714.95	89.494.97	0.0005***
Kim-1 (*Havcr1*) mRNA expression *(Fold Change)*	0.960.20	278.00126.60	0.0022**
Ki67 (*Mki67*) mRNA expression *(Fold Change)*	1.060.14	4.041.20	0.0012**

**Parameters**	**CTL (*n* = 4–6)**	**NaOx (*n* = 4–7)**	***p*-value**

Relative megalin protein expression	6.130.65	1.850.87	0.0286*
Corrected tubular fluorescence for megalin	5.730.73	1.870.27	0.0026**
Relative THP expression	1.090.11	0.590.07	0.0082**

**Parameters**	**CTL (*n* = 5–6)**	**NaOx (*n* = 5–8)**	***p*-value**

CD68 positive cells/field	6.801.80	24.003.97	0.0065**
MCP-1 (*Ccl2*) mRNA expression *(Fold Change)*	1.020.08	3.160.43	0.0007***
IL-1β (*Il1b*) mRNA expression *(Fold Change)*	0.950.19	2.440.37	0.0159*
NFkappaB mRNA expression *(Fold Change)*	1.000.03	1.410.11	0.0043**
α-SMA (*Acta2*) mRNA expression *(Fold Change)*	1.010.07	1.531.12	0.0047**

Values are mean ± SE, number of animals (n) per group is indicated in parenthesis. *p < 0.05, **p < 0.01, ***p < 0.001 versus control (CTL); NaOx, sodium oxalate. Alb/Cr, albumin/creatinine; A. U, arbitrary units; THP, Tamm–Horsfall protein.

### Glomerular Injury

**FIGURE 2:**
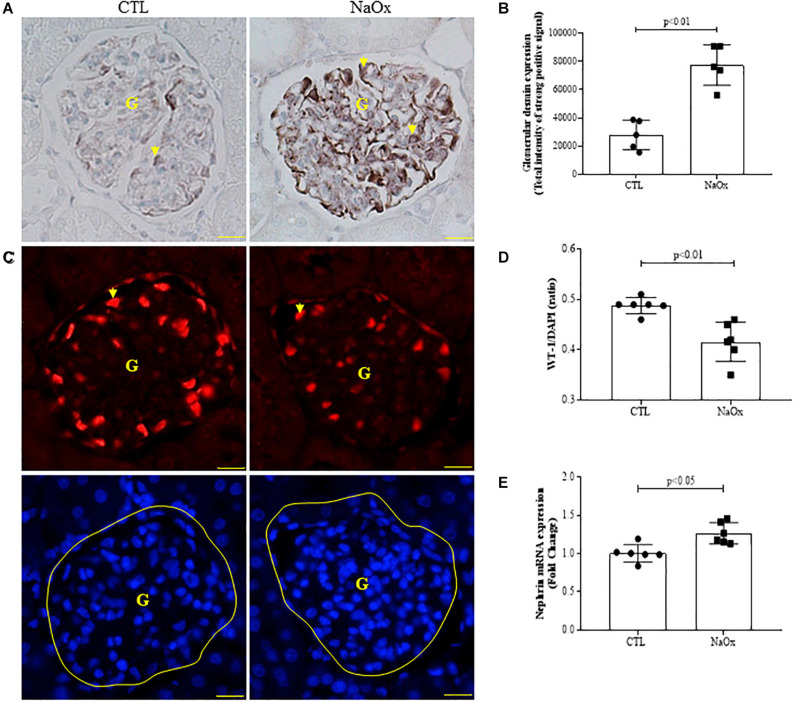
Effect of NaOx treatment on desmin staining **(A,B)**, WT1/DAPI ratio **(C,D)**, and nephrin mRNA expression **(E)**. The values represent the mean ± SE (*n* = 5–6/group). Images were captured using a morphometric program (NIS-Elements, Nikon) with a 40× objective. The arrows indicate desmin and WT1 staining, and G indicates the glomerulus. Bar = 50 μm. CTL, control; NaOx, sodium oxalate.

Next, we evaluated glomerular conditions in both the control and treated groups. NaOx treatment induced a significant increase in glomerular desmin immunostaining (Figures 2A,B), decreased the WT1 immunofluorescence signal (Figures 2C,D) and increased nephrin mRNA expression (Figure 2E) in comparison to the control group. The mean values are shown in Table 2.

### Tubular Injury

**FIGURE 3:**
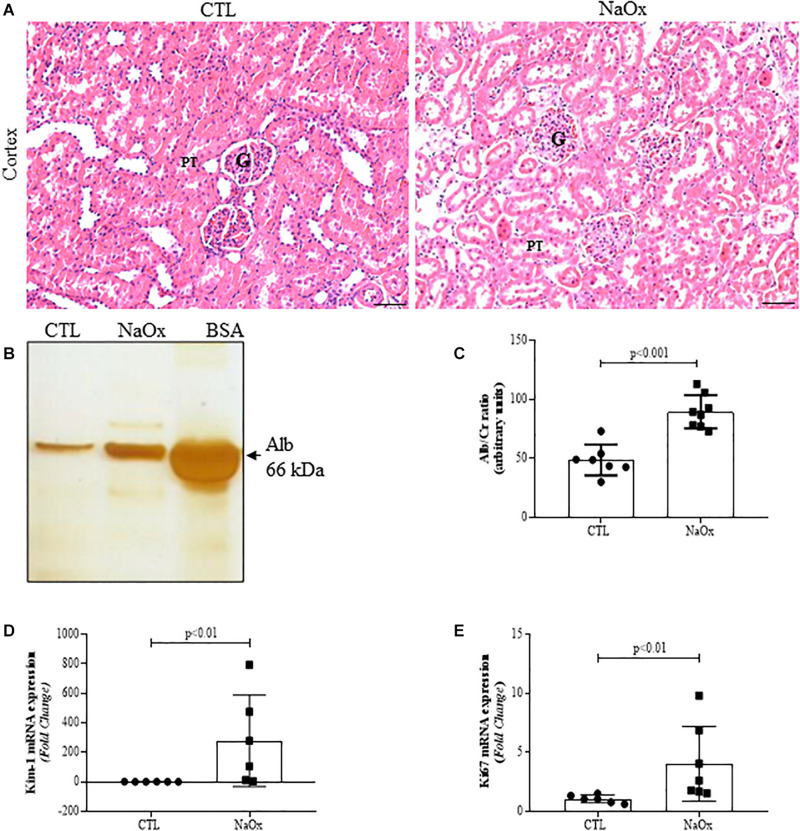
Effect of NaOx treatment on cortical changes, including tubular injury, as demonstrated by HE staining **(A)**. Urine samples from control and treated rats were resolved on 10% SDS page gels followed by gel staining with a protein-sensitive SilverQuest Silver Staining Kit. Statistical analysis of albumin excretion in both the control and treated groups was performed **(B,C)**. The mRNA expression levels of Kim-1 **(D)** and Ki67 **(E)**. The values are mean ± SE (*n* = 6–8). Bar = 100 μm. CTL, control; NaOx, sodium oxalate; G glomerulus; PT, proximal tubule.

To determine if treatment with NaOx induces morphological changes in the kidneys, 4-μm hematoxylin and eosin-stained kidney sections were evaluated, and the results indicated that there was prominent tubular dilation in the treated group in comparison to the control group (Figure 3A). Furthermore, NaOx treatment induced albuminuria (Figures 3B,C) and a significant increase in Kim-1 and Ki67 mRNA expression (Figures 3D,E). The mean values are shown in Table 2.

**FIGURE 4:**
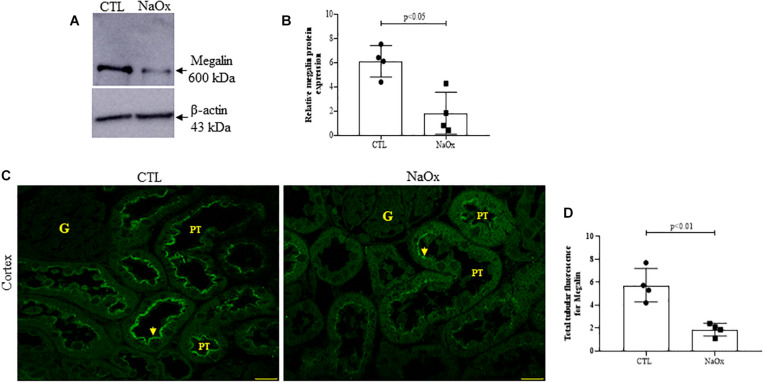
Effect of NaOx treatment on total megalin protein expression. **(A,B)** Immunoblot analysis was performed on 50-μg protein aliquots resolved by 4% SDS-PAGE. The values are presented as the mean ± SE (*n* = 4/group) and β-actin was used as an internal control. Megalin staining in the proximal tubule (PT) **(C)** is indicated by the arrows. Images of megalin immunofluorescence were captured using a Zeiss LSM 510 confocal microscope equipped with a 63× objective. Statistical analysis of megalin immunofluorescence in both the control and treated groups was performed **(D)**. CTL, control; NaOx, sodium oxalate; G, glomerulus. Bar = 50 μm.

Tubular injury in the NaOx-treated group occurred concomitantly with a decrease in total megalin protein expression and proximal tubular megalin distribution in comparison to those observed in the control animals (Figures 4A–D). The mean values are shown in Table 2.

### THP Expression

**FIGURE 5:**
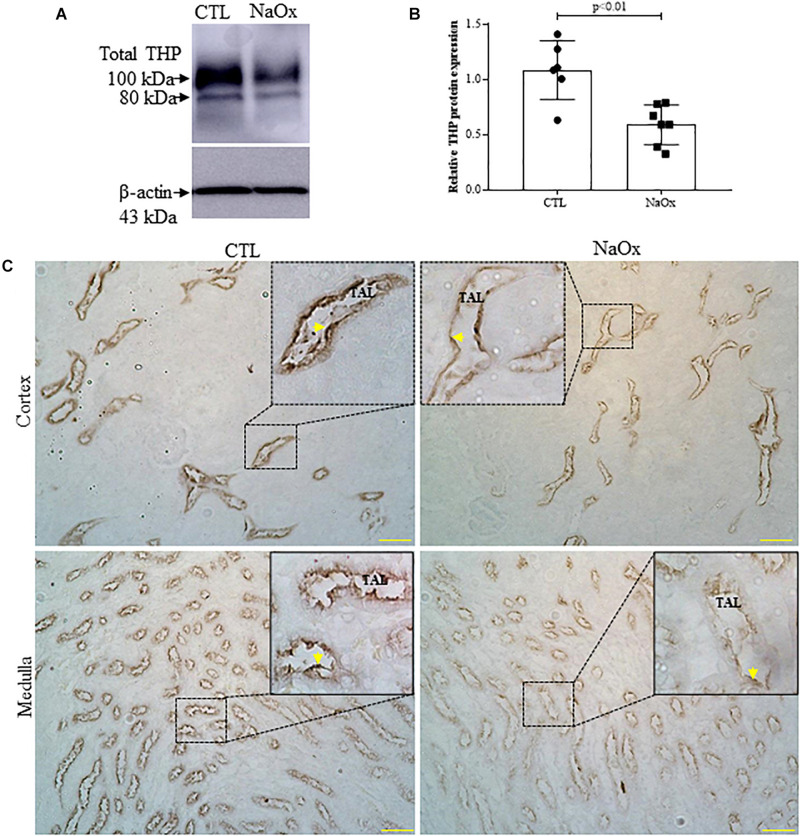
Tamm–Horsfall protein (THP) expression and distribution. Total THP expression in the kidney **(A,B)**. Immunoblot analysis was performed on 50-μg protein aliquots resolved by 10% SDS-PAGE. The values represent the mean ± SE (*n* = 6–7/group) and β-actin was used as an internal control. **(C)** Immunohistochemical images of THP staining in the cortex and medulla. Standardized images were captured using a morphometric program (NIS-Elements, Nikon) with a 20× objective and magnified images captured with a 40× objective are shown at the top. The arrows indicate THP staining in the cortex and thin ascending limb (TAL). Bar = 100 μm. CTL, control; NaOx, sodium oxalate.

Since distal nephron segments showed protein casts in the treated group, as reported before, we investigated THP expression and its tubular distribution. The results demonstrated that NaOx treatment induced a significant decrease in the total of THP protein expression and distribution in both cortical and medullary regions of the kidney in comparison to those observed in the control group (Figures 5A–C and Table 2).

### Proinflammatory and Profibrotic Factors

**FIGURE 6:**
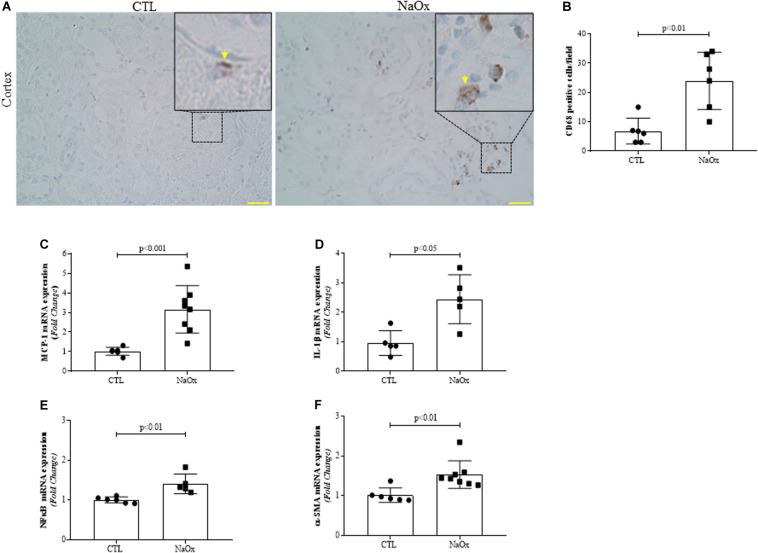
Representative photomicrographs of the renal cortex illustrating tubulointerstitial CD68-positive cells (indicated by arrows) by immunohistochemistry. Images were captured using a morphometric program (NIS-Elements, Nikon) with a 20× objective and magnified images shown at the top were captured with a 40× objective. The values represent the mean ± SE **(A,B)**. Bar = 100 μm. In addition, MCP-1 **(C)**, IL-β1 **(D)** NFkappaB **(E)** and α-SMA **(F)** mRNA expression levels are shown as the mean ± SE (*n* = 5–8/group). CTL, control; NaOx, sodium oxalate.

We performed immunohistochemistry for CD68 (ED1), a monocyte/macrophage marker. Treatment with NaOx induced a significant increase in CD68 protein staining, indicating the infiltration of macrophages in the kidney (Figures 6A,B). Next, we investigated the gene expression of the proinflammatory and profibrotic factors. We observed that NaOx treatment induced a significant increase in MCP-1, IL-1β, NFkappaB, and α-SMA mRNA expression, in comparison to that in the control group (Figures 6C–F and Table 2).

## Discussion

In the current study, a single intraperitoneal injection of NaOx in male rats induced CaOx crystal and luminal cast formation in the medullary tubules, observed 24 h after treatment. This experimental model was characterized by Khan et al. (1979, 1982, 1992), who reported an increase in urinary excretion of oxalate after a single NaOx injection, followed by its decrease within the next 12 h. In our study we observed a still prominent excretion of crystals in 24 h. Here, we focused on the factors involved in crystalline-induced AKI and consequent changes in renal function and morphology in 24 h.

In our study, although the metabolic parameters were similar between the groups, we observed a significant decline in kidney function in rats treated with NaOx, since under this condition the animals showed increased plasma level of creatinine followed by reduced creatinine clearance. It is known that serum creatinine level is a suboptimal marker of glomerular injury, since it depends of many factors, including the new glomerular filtration rate (GFR), tubular secretion rate and urine volume (Moran and Myers, 1985; Arai et al., 2016). Thus, in the current study we also evaluated different factors related to glomerular injury. Our results demonstrate increased plasma level of urea, enhanced glomerular desmin staining intensity, decreased podocyte WT1 staining, increased nephrin mRNA expression in the NaOx-treated group. Together, our results indicate consistent glomerular injury in crystalline-induced AKI. Our findings corroborate other studies that demonstrated that tubular casts formed by crystals and/or necrotic cell debris can transiently obstruct the tubular lumen, resulting in decline of glomerular filtration rate (GFR) (Arai et al., 2016). Podocytes are terminally differentiated and non-proliferative glomerular epithelial cells that are situated on the outer surface of the glomerular capillary basement membrane (GBM) (Qian et al., 2019). Under healthy conditions, podocyte foot processes are connected to each other by slit diaphragms (SDs), which are organized by cross-linking molecules such as nephrin, podocin, Neph1, CD2AP and cytoskeleton proteins (Faul et al., 2007; Feliers, 2015; Garg, 2018). In addition, WT1 is a transcription factor that regulates the differentiation state of podocytes and is highly expressed in mature podocytes (Guo et al., 2002). It has been documented that injured podocytes re-enter the cell cycle, leading to dedifferentiation, podocyte damage and a loss of ultrafiltration barrier function, resulting in proteinuria (Greka and Mundel, 2012), which is the major risk factor for progressive chronic kidney disease. The increased nephrin mRNA expression observed in our study suggests a compensatory strategy of podocytes against the possible loss of damaged cells.

In healthy glomeruli, desmin is distributed mainly in mesangial cells. However, in different experimental models of mice and rats with glomerular disease, increased glomerular desmin staining is recognized as a marker of podocyte dedifferentiation and injury (Crowley et al., 2009; Casare et al., 2016; Xie et al., 2019) and is followed by decreased nuclear WT1 expression and loss of slit diaphragm proteins (Greka and Mundel, 2012).

Considering the relevance of podocyte injury to progressive proteinuria previously demonstrated in ischemia-reperfusion and glomerulosclerosis animal models (Hara et al., 2015; Chen et al., 2019), we also evaluated whether acute crystalline-related glomerular injury contributes to proteinuria. Indeed, we observed significant albuminuria in treated animals in comparison to the control group. However, this result was not exclusively attributable to podocyte injury since we found increased tubular injury factors mRNA expression, as discussed below. Thus, we extended our observations from the glomerulus to the renal tubules.

It is established that in kidney crystalline diseases, intraluminal crystals deposition leads to tubular obstruction, followed by crystals endocytosis through tubular cells. Under these conditions, sensitized tubular cells release damage-associated molecular patterns (DAMPs) to the interstitial compartment. DAMPs activate proteins such as NACHT, LRR and PYD domains-containing protein 3 (NLRP3) inflammasome in renal dendritic cells. In addition, intraluminal crystals can be translocated to the interstitial compartment, where are phagocytized by dendritic cells and macrophages, resulting in activation of NLRP3 inflammasome and consequent secretion of IL-1β (Mulay et al., 2013; Mulay and Anders, 2017). In turn, IL-1β induces numerous inflammatory responses.

Relative to proximal tubule (PT), its epithelium has a remarkable capacity to repair itself since it has potent ability to replace lost cells through proliferation (Bonventre, 2003). Notably, proximal tubule cells express kidney injury molecule 1 (Kim-1), which is highly upregulated after acute kidney injury (Ichimura et al., 1998; Sabbisetti et al., 2014), acts as a marker of cell differentiation and proliferation (Han et al., 2002), and mediates the phagocytosis of oxidized lipids and apoptotic bodies, including luminal cellular debris (Ichimura et al., 2008). It has been established that in addition to Kim-1, Ki67 is used as a marker of tubular regeneration and renal repair after AKI (Lazzeri et al., 2018; Zhou et al., 2018). Severe AKI could result in incomplete repair and a persistent increase in Kim-1 and Ki67 expression in tubular cells leads to AKI-to-CKD transition (Dong et al., 2019). In the current study, the proximal tubular injury was associated to a decreased megalin protein expression. Megalin is a large glycoprotein (∼600 kDa) expressed mainly in PT cells that acts as an endocytic receptor; it accumulates in clathrin-coated pits and is involved in the reabsorption of various molecules, including albumin and other low-molecular-weight proteins from glomerular filtrates (Cui et al., 1996).

We also evaluated whether THP is involved in crystalline-induced AKI. THP, or uromodulin, is a multifunctional renal-specific glycoprotein produced by the epithelial cells of the thick ascending limb (TAL) of Henle’s loop, targeted to the apical domain through its glycosylphosphatidylinositol (GPI) anchor site and subsequently cleaved by the serine protease and released in the lumen (Brunati et al., 2015). In the urine, THP tends to assemble in multimeric networks through its Zona Pellucida (ZP) domain to form a dense matrix of high-molecular weight polymers constituting hyaline casts, which are increased in AKI (Devuyst et al., 2017). Although predominantly secreted into the urine, THP is also released at the basolateral membrane as a monomeric form toward the interstitial compartment, where it mediates a protective crosstalk between TAL and S3 segments of the proximal tubules, downregulating inflammatory cytokines such as MCP-1and TNF-α mainly during AKI recovery (Safirstein et al., 1991; Rampoldi et al., 2011; Heitmeier et al., 2014). El-Achkar group and other investigators using renal ischemia-reperfusion injury (IRI), but not crystalline-experimental models, observed that the THP mRNA and protein expression were reduced at the peak of injury (24 h), and recovered 72 h and 6 days after the surgery (Safirstein et al., 1991; El-Achkar et al., 2008; El-Achkar et al., 2013; Micanovic et al., 2018). Our results corroborate these findings, since we observed a relevant decrease in total THP protein expression in the kidney as well as a decrease in tubular THP protein staining in crystalline-induced AKI.

It is known that in crystalline-induced AKI, a rapid and diffuse crystallization contribute to kidney injury in several ways, including crystal-induced cytotoxicity triggers inflammation (Mulay and Anders, 2017). However, the mechanisms involved in these processes have not been elucidated. Considering the macrophage phenotypes, it is well described that pro-inflammatory M1 macrophage releases cytokines such as IL-1, IL-6, TNF-α, and MCP-1, which in turn promotes macrophage recruitment and activation during kidney injury (Kanda et al., 2006; Ricardo et al., 2008; Tesch, 2008; Dias et al., 2017). In addition to activating macrophages, IL-1β can also activate NFkappaB p65 (Shi and Berger, 2018). Activated NFkappaB p65 exacerbate the transcription of inflammatory mediators such IL-1β and MCP-1 (Anders, 2016). In contrast, M2 macrophages seems to be anti-inflammatory and profibrotic, secreting IL-10, fibronectin, TGF-β and other ECM proteins, triggering the accumulation of myofibroblasts that express smooth muscle α-actin (αSMA) (Tang et al., 2019). Consistent with these findings, our results revealed that crystalline-induced AKI resulted in an increased expression of interstitial CD68, a macrophages marker, MCP-1, IL-1β and NFkappaB mRNA, suggesting a relevant signal crosstalk between inflammatory response and cellular injury. In addition to inflammatory condition, we found profibrotic signal, highlighting the contribution of both macrophage subtypes in crystalline-induced AKI.

## Future Perspective and Directions for Research

It is now becoming clear that in crystalline-induced AKI, inflammatory factors contribute to renal pathology, including a possible crosstalk between tubulointerstitial compartment and the glomerulus. However, considering the acute condition of the current study, we cannot confirm all biological events related to glomerular injury. On the other hand, our data are relevant and open perspectives for understanding of the crystalline-induced CKD. A deeper knowledge about the temporal events in the kidney will certainly provide a better understanding of crystalline nephropathies.

## Conclusion

Renal pathology is the result of different cellular programming pathways, whose responses involve risks that depend on the host defense and tissue repair upon injuries. Taken together, our results suggest that acute crystalline nephropathy induces glomerular injury with a loss of podocyte viability in addition to strong tubulointerstitial injury. Thus, we believe that our findings will contribute to understanding of crystal biology and help to improve patient outcomes by defining novel cellular and molecular targets to limit nephron loss and to maintain renal function.

## Data Availability Statement

The raw data supporting the conclusions of this article will be made available by the authors, without undue reservation, to any qualified researcher.

## Ethics Statement

The animal study was reviewed and approved by Institutional Animal Care and Use Committee of the University of São Paulo (Protocol no. 9276140518).

## Author Contributions

LA designed and performed all the experiments, analyzed the data, helped to writing, and reviewed the manuscript. JC-P helped with animals treatment, metabolic parameters analysis, immunoblotting, immunohistochemistry, immunofluorescence, and mRNA expression experiments. MP helped with animal treatments, kidney function analysis, and gene and protein expression experiments. MO-S supported and supervised the study and contributed with the writing of the manuscript. All authors approved the final manuscript.

## Conflict of Interest

The authors declare that the research was conducted in the absence of any commercial or financial relationships that could be construed as a potential conflict of interest.
